# Clinical profile of vitiligo patients and relationship with immuno-inflammatory markers^☆^

**DOI:** 10.1016/j.abd.2023.03.007

**Published:** 2023-11-18

**Authors:** Marta Regina Machado Mascarenhas, Mariana de Castro Oliveira, Luise Fonseca de Oliveira, Andréa Santos Magalhães, Paulo Roberto Lima Machado

**Affiliations:** Dermatology Service, Hospital Universitário Professor Edgard Santos, Salvador, BA, Brazil

**Keywords:** Autoimmunity, Biomarkers, Chemokines, Cytokines, Vitiligo

## Abstract

**Background:**

Vitiligo is the most common pigmentary disorder and is considered a chronic, cumulative, multifactorial disease. The crucial role of cytotoxic CD8+ T lymphocytes and the IFNγ/CXCL10 axis has been demonstrated in its pathogenesis.

**Objective:**

To evaluate the clinical profile and immuno-inflammatory markers in patients with vitiligo in a reference medical center.

**Methods:**

Cross-sectional study in which all patients with vitiligo seen at the medical center the from 2019 to 2022 were evaluated, to outline the clinical profile. Moreover, cardiovascular risk biomarkers (neutrophil/lymphocyte ratio and C-reactive protein levels) were measured, as well as cytokines and chemokines (TNFα, IFNγ, IL10, IL15 and CXCL10) in the serum of a subgroup of 30 patients.

**Results:**

There was a predominance of females, with a mean age of 43 years. Most were phototypes IV or V (71.3%), without comorbidities (77.55%), and without a family history of vitiligo (70.41%). Higher levels of neutrophil/lymphocyte ratio, C-reactive protein, and inflammatory cytokines/chemokines were documented in vitiligo patients when compared to the control group (non-significant). As relevant data, the highest values of CXCL10 were detected in patients with vitiligo *versus* controls, as well as in patients with disease of shorter duration (p < 0.05).

**Study limitations:**

The number of assessed patients was small due to recruitment difficulties caused by the COVID-19 pandemic.

**Conclusion:**

The present data contribute to confirming the relevant role of the IFNγ/CXCL10 axis in the pathogenesis of vitiligo, highlighting CXCL10 as a possible activity marker.

## Introduction

Vitiligo affects 0.5% to 2% of the world population and its key event is the destruction of melanocytes in the affected skin. The mean age of onset is 20 years, with 95% of cases typically occurring before 40 years of age.^1^, ^2^, ^3^

The pathogenesis of vitiligo has the following theories as its main hypotheses: neural, autoimmune and oxidative stress. None of these theories, alone, can explain vitiligo in its entirety.^4^ Vitiligo has been described as an autoimmune disease with genetic, environmental, metabolic and oxidative factors associated. It is likely that these factors mix in a heterogeneous way, contributing to the different presentations of the disease.^5^

Vitiligo is clinically characterized by depigmentation of the skin and hair follicles, commonly beginning in areas of trauma (Koebner phenomenon).^6^ The disease usually spares melanocytes in the hair follicle because of the immune context there, similar to other sites that also contain melanocytes, such as the brain, eyes, and inner ear. Areas with white hair or no hair are more difficult to recover.^7^

Vitiligo can be classified into segmental and non-segmental.^2^ The segmental forms usually appear in childhood and are characterized by unilateral achromic patches in linear or block patterns, with early leukotrichia and poor response to treatments.^5^, ^6^, ^8^, ^9^ Non-segmental forms can be localized, generalized, universal and mixed. The localized forms are subdivided into focal and mucosal. Focal vitiligo is characterized by a small achromic spot (10‒15 cm^2^). Mucosal vitiligo occurs when at least one site of involvement is the oral or genital mucosa. The acrofacial variants are localized forms that are more resistant to treatments. The generalized forms present achromic macules in different parts of the body (affected body area < 80%) and in the universal forms, there is complete or almost complete depigmentation of the skin (involvement >80%). The mixed form may present with lesions compatible with segmental and non-segmental vitiligo.^2^, ^6^

The psychological impact is important,^10^, ^11^ with anxiety being a common symptom, which affects the social and sexual lives of these patients.^1^ Although it is a disease with predominantly cutaneous involvement, new studies show systemic involvement, with a higher frequency of insulin resistance, metabolic syndrome, cardiovascular and autoimmune diseases.^11^, ^12^, ^13^, ^14^

Recent studies have highlighted the role of inflammatory chemokines, such as CXCL9, CXCL10, and CXCL12.^15^, ^16^ These chemokines are related to the Th1 response and are important in the process of recruitment and activation of cytotoxic CD8+ T lymphocytes.^17^, ^18^ In inflamed tissues, lymphocytes optimize the production of IFNγ and TNFα, which, in turn, stimulate CXCL10 secretion. This chemokine binds to its receptor on the cells (CXCR3) and triggers an inflammatory cascade with activation and greater recruitment of T-lymphocytes, contributing to melanocyte destruction. CXCL10 is also increased in other autoimmune diseases, such as lichen sclerosus and atrophicus, autoimmune thyroiditis, Graves' disease, alopecia areata, and rheumatoid arthritis.^19^

The autoimmune theory has been reinforced by the following more recent findings^13^, ^14^: first, CD8+ T-lymphocytes and the IFNγ axis seem to play a crucial role in inhibiting melanogenesis and inducing melanocyte apoptosis,^20^ and second chemokines such as CXCL10 may play a role in the progression and maintenance of vitiligo.^21^

However, there are still many gaps regarding immunological and inflammatory parameters which hamper a better understanding of its pathogenesis and the development of more effective therapeutic strategies.

## Methods

This was a cross-sectional study with vitiligo patients from the dermatology service, aimed at evaluating the clinical profile, cytokines and inflammatory parameters. To characterize the clinical profile, between February 2019 and January 2022, all registered patients aged between 18 and 60 years (total of 98) were assessed. The patient database included information on identification, disease data (time of onset, personal or family history of vitiligo or other autoimmune diseases, previous treatments), type of vitiligo, extent of the disease, and prescribed treatments. To evaluate the inflammatory and immunological parameters, 30 patients were recruited from March 2021 to January 2022. The selection was carried out by spontaneous demand of those patients seen at the service the service and who were previously registered in the database, randomly and after consent.

### Clinical evaluation

Patients were evaluated by a single dermatologist who classified the clinical form of vitiligo as well as the extent of the disease according to the VETI (Vitiligo Extent Tensity Index).^22^ The VETI score calculation comprises: (percentage of head involvement × stage of depigmentation) + (percentage of trunk involvement × stage of depigmentation) 4 + (percentage of upper limb involvement × stage of depigmentation) 2 + (percentage of lower limb involvement × stage of depigmentation) 4 + (percentage of genital involvement × stage of depigmentation) 0.1. The maximum value of the VETI score is 55.5.

### Laboratory evaluation

Blood count and CRP (C-Reactive Protein) data for the subgroup of 30 patients were obtained from the medical records from the last consultation, with a maximum period of three months before the measurement of cytokine/chemokine levels. The NLR (Neutrophil/Lymphocyte Ratio) was calculated by dividing the absolute number of neutrophils by the number of lymphocytes, obtained from the blood count. Cytokines and chemokines of ten healthy controls were measured and used as a comparison group.

To measure cytokine/chemokine levels (TNFα, IFNγ, IL10, IL15, CXCL10), 10 mL of blood was collected and processed using the ELISA technique. For all measurements, BD Bioscience kits (BD OptEIA*^TM^* Set) were used. Results were calculated using the InStat program (GraphPad, San Diego CA) and expressed in pg/mL. Patients who refused to participate, patients with active viral or bacterial diseases, those under systemic immunosuppressive therapy in the 30 prior days, and patients with other autoimmune diseases such as thyroid disease, rheumatoid arthritis, and systemic lupus erythematosus were excluded.

### Statistical analysis

Clinical and laboratory data were analyzed using the free software R (R version 4.1.1 [2021-08-10]). The *p* value was considered significant when < 0.05. Quantitative variables were summarized in descriptive tables using mean and standard deviation. To compare variables between groups, a one-way ANOVA test and Student's t-test were used for categorical variables that followed a normal distribution. To compare the categorical variables that did not follow the normality pattern, the Kruskal-Wallis and Mann-Whitney tests were used. Spearman's correlation was used to compare interval quantitative variables that did not follow this pattern.

### Ethical aspects

The study was conducted in accordance with the guidelines of Resolution N. 466/2012, having been approved by the Ethics and Research Committee (Counsel number 4,886,709).

## Results

As shown in Table 1, it can be observed that the mean age of patients was 43.3 years, with a predominance of females (78.6%). Phototype IV or V was present in 71.3%, with phototype IV being more common (43.8%). The most common vitiligo subtype was generalized (54%), followed by focal (17.3%) and acrofacial (10.2%). When assessing the extent of the disease, a mean VETI score of 3.11 was observed.^22^ In the sample of 30 participants, the mean age was 45.2 years. Females (86.6%), phototype IV (43.3%), and the generalized subtype (63.3%) prevailed, data similar to what was found in group of 98 participants, with no statistically significant differences between the groups. More than 70% of the respondents had no comorbidities or a family history of vitiligo. The most commonly associated autoimmune disease was hypothyroidism, followed by hyperthyroidism.

**Table 1:** Clinical profile of patients with vitiligo at the dermatology outpatient clinic.

Study population	Group A^a^ (n = 98)	Group B^b^ (n = 30)
Age	43.3 (±18.2)	45.2 (±10.7)
Gender		
Female	77 (78.6%)	26 (86.6%)
Male	21 (21.5%)	4 (13.4%)
Phototype		
III	13 (13.2%)	3 (10%)
IV	43 (43.8%)	13 (43.3%)
V	27 (27.5%)	10 (33.3%)
VI	15 (15.31%)	4 (13.3%)
Duration of disease (years)	16.3 (±14.6)	16 (±13.3)
Comorbidities	22 (22.4%)	8 (26.7%)
Family history	29 (29.6%)	7 (23.3%)
Clinical type		
Generalized	53 (54.1%)	19 (63.3%)
Focal	17 (17.3%)	1 (3.3%)
Acrofacial	10 (10.2%)	6 (20%)
Segmental	9 (9.2%)	2 (6.7%)
Universal	9 (9.2%)	2 (6.7%)
VETI	3.11 (±8.52)	3.36 (±9.35)

VETI (Vitiligo Extent Tensity Index), score that assesses the extent of the disease.

a Group A: comprises the total number of patients followed in the period.

b Group B: comprises the number of patients submitted to analysis of immune response and risk markers.

Table 2 shows the mean and standard deviation of the values obtained for the immunoinflammatory markers measured in a sample of 30 participants and 10 healthy controls. The localized group included the focal, acrofacial, and segmental forms, whereas the generalized group included the generalized and universal forms. Student’s t and one-way ANOVA tests were performed for the markers that followed the normality pattern (NLR and CXCL10) and for the others, the Mann-Whitney and Kruskal-Wallis tests (p values in the table).

**Table 2:** Immuno-inflammatory markers and type of vitiligo (subgroup of 30 participants and 10 healthy controls).

Marker	Controls (n = 10)	Localized vitiligo (n = 9)	Generalized vitiligo (n = 21)	p-value
**NLR (n = 30)**				
Mean (SD)	–	2.1 (±0.8)	2.33 (±0.9)	0.5
**CRP (n = 24)**				
Mean (SD)	–	2.3 (±3.43)	3.2 (±5.3)	0.4
**TNFα**				
Mean (SD)	6.1 (±6.8)	10.6 (±11.7)	3.9 (±6.5)	0.2
**IFNγ**				
Mean (SD)	23.7 (±15.5)	16.7 (±4.4)	22.4 (±12.8)	0.4
**IL10**				
Mean (SD)	23.1 (±1.2)	25.6 (±5.4)	24.3 (±3.3)	0.7
**IL15**				
Mean (SD)	13.1 (±3.78)	14.1 (±7.4)	12.6 (±4.1)	0.7
**CXCL10**				
Mean (SD)	131.2 (±77.4)	186.3 (±110.5)	182.6 (±94)	0.3

NLR, Neutrophil/Lymphocyte Ratio; CRP, C-Reactive Protein.

**Figure 1:**
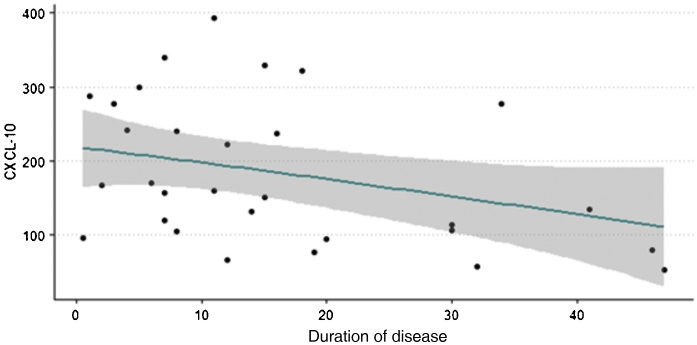
Relationship between serum levels of CXCL10 and duration of vitiligo (years).

Fig. 1 shows that higher levels of chemokine CXCL10 are observed in patients with vitiligo of shorter duration. Spearman's correlation test was performed with a p-value < 0.05, indicating an inverse relationship between the variables.

Analyses were made between NLR, CRP, levels of TNFα, IFNγ, IL10 and IL15, and the duration of the disease. All of them showed a trend towards higher values in patients with disease of shorter duration; however, the p-value was not statistically significant.

## Discussion

There was a higher prevalence of vitiligo among women (78.5%), a finding compatible with the meta-analysis carried out by Zhang et al.^23^; however, that was different from other previous studies, in which the prevalence between men and women was shown to be equal.^24^, ^25^ This result is probably due to the greater cultural concern with skin depigmentation in females. The mean age found in the present study, 43 years, differs from this same meta-analysis, in which the prevalence was more common in patients over 60 years.^23^ This divergence may have occurred due to the lower access by the elderly during the COVID-19 pandemic. According to the Fitzpatrick scale, most patients were phototypes IV and V (71.3%). In the Brazilian study by Cerci et al. (2010),^26^ there was a predominance of phototype IV. The mean duration of the disease was greater than 15 years, demonstrating the its chronicity.

Most patients had no comorbidities or family history of vitiligo. Epidemiological studies show that cases of vitiligo tend to be more common in the same family; however, the genetic risk is not absolute.^5^ When there is a history of the disease in a first-degree relative, the risk of developing vitiligo is 6%.^27^

More recent data have shown that vitiligo may be associated with increased cardiovascular risk, insulin resistance, and metabolic syndrome,^12^, ^28^ as disclosed in the study of 96 patients by Karadag et al. (2011),^29^ where patients with vitiligo had a higher prevalence of insulin resistance, higher LDL levels, and lower HDL levels. The association of vitiligo with juvenile DM, type 2 DM, acanthosis nigricans, and as a component of the HAIR-AN (hyperandrogenism, insulin resistance, and acanthosis nigricans) syndrome has been described.^30^, ^31^, ^32^ Interestingly, the melanocytes present in adipose tissue seem to play an important role in neutralizing oxygen-free radicals.^12^, ^28^, ^33^ Thus, the concept of a purely cutaneous disease with a psychological impact due to cosmetic disfigurement is questioned, in favor of a broader thesis, that identifies it as an auto-inflammatory disease with systemic implications.

There are few studies that evaluated systemic inflammatory markers in vitiligo. The neutrophil/lymphocyte ratio (NLR) has been considered a marker of increased cardiovascular risk. Solak et al. (2017)^12^ concluded that NLR is related to endothelial dysfunction, has prognostic value, and could predict independent cardiovascular outcomes. The present data showed that the mean patient NLR was 2.24, similar to the mean found in vitiligo patients in the study by Solak et al. (2017).^12^ Higher NLR values were observed in patients with generalized vitiligo when compared to those with localized disease. However, as the sample was small, the present data was not significant, but consistent with the hypothesis that associates extensive disease with systemic implications.

In the present study, the mean C-Reactive Protein (CRP) level was 2.92 mg/dL. Badimon et al. (2018)^34^ showed that levels >1 mg/dL were considered as medium cardiovascular risk and >3 mg/dL as high risk. Regarding CRP levels and type of vitiligo, a trend towards higher values was also observed in generalized cases, as demonstrated by Solak et al. (2017).^12^

Although the cytokine and chemokine levels in the peripheral blood of healthy controls and vitiligo patients subdivided according to type do not show statistically significant differences (perhaps due to the sample size), only CXCL10 is clearly increased in vitiligo patients when compared to controls.

A relevant finding of this study is the inverse correlation between chemokine CXCL10 levels and duration of the disease; the highest values are found in patients with shorter disease duration, that is, those in the most initial and acute phase, corroborating the results by Wang et al. (2016).^35^ Active and progressing vitiligo is often more common in patients with a shorter evolution.^17^ This trend was also observed in the measurements of other cytokines (TNFα, IFNγ, IL10 and IL15), despite the lack of statistical significance. Higher levels of CXCL10 were also observed in patients with acrofacial and generalized forms, when compared to patients with universal forms, consistent with the study by Gharib et al. (2021).^17^ It is possible that the clinical stability of universal vitiligo may explain this finding.

Patient recruitment was hindered by the COVID-19 pandemic, as accessibility and care were reduced for a period of two years. Thus, the sample was small for comparative analyses, but it allowed obtaining a clinical profile of vitiligo patients in this reference service and important data, corroborating the role of CXCL10 in disease pathogenesis.

Vitiligo is an autoimmune disease with an important participation of cytotoxic TCD4+ and TCD8+ lymphocytes.^17^, ^36^, ^37^ There is an increase in cytotoxic T lymphocytes, both in peripheral blood and in skin lesions. In the skin these cells infiltrate the epidermis and accumulate around the melanocytes.^17^

The literature emphasizes the role of inflammatory chemokines such as CXCL9, CXCL10 and CXCL12, with emphasis on CXCL10.^15^, ^16^ The production of CXCL10 is regulated by IFNγ (secreted by CD8+ lymphocytes), which induces the transcription of factors via NF-kB, Janus Kinase and STAT1 (JAK/STAT), activating the signaling cascade.^38^ This activation stimulates greater production of CXCL10, leading to a cycle that feeds itself. This IFNγ pathway is also important in maintaining existing lesions.^7^ This suggests that the most promising treatments might involve not only blocking CXCL10 and the IFNγ pathway, but also controlling or destroying resident memory T-cells. In this line of work, JAK inhibitors that block the IFNγ signaling pathway have shown to be promising for some dermatological diseases such as atopic dermatitis, alopecia areata, and vitiligo.^39^ More recently, topical JAK inhibitors, such as tofacitinib (JAK1/3 inhibitor) and ruxolitinib (JAK1/2 inhibitor), have been used with some success in vitiligo.^40^

## Conclusion

The present study contributes to confirming a relevant role of the IFNγ/CXCL10 axis and, consequently, of the JAK/STAT pathway in the pathogenesis of vitiligo, highlighting CXCL10 as a possible marker of disease activity.^21^, ^36^

## Financial support

None declared.

## Authors' contributions

Marta Regina Machado Mascarenhas: Statistical analysis; design and planning of the study; drafting and editing of the manuscript; collection, analysis, and interpretation of data; intellectual participation in the propaedeutic and/or therapeutic conduct of the studied cases; critical review of the literature.

Mariana de Castro Oliveira: Drafting and editing of the manuscript; collection, analysis, and interpretation of data.

Luise Fonseca de Oliveira: Drafting and editing of the manuscript; collection, analysis, and interpretation of data.

Andréa Santos Magalhães: Collection, analysis and interpretation of data.

Paulo Roberto Lima Machado: Approval of the final version of the manuscript; design and planning of the study; drafting and editing of the manuscript; effective participation in research orientation; intellectual participation in the propaedeutic and/or therapeutic conduct of the studied cases; critical review of the manuscript.

## Conflicts of interest

None declared.
